# A Novel Fatty Acid Metabolism-Associated Risk Model for Prognosis Prediction in Acute Myeloid Leukaemia

**DOI:** 10.3390/curroncol30020193

**Published:** 2023-02-20

**Authors:** Nana Wang, Xiaoran Bai, Xinlu Wang, Dongmei Wang, Guangxin Ma, Fan Zhang, Jingjing Ye, Fei Lu, Chunyan Ji

**Affiliations:** 1 Department of Hematology, Qilu Hospital of Shandong University, Jinan 250012, China; 2 Hematology and Oncology Unit, Department of Geriatrics, Qilu Hospital of Shandong University, Jinan 250012, China; 3 Department of Critical Care Medicine, Qilu Hospital of Shandong University, Jinan 250012, China

**Keywords:** fatty acid metabolism, prognosis signature, acute myeloid leukaemia

## Abstract

Acute myeloid leukaemia (AML) is the most common acute leukaemia in adults, with an unfavourable outcome and a high rate of recurrence due to its heterogeneity. Dysregulation of fatty acid metabolism plays a crucial role in the development of several tumours. However, the value of fatty acid metabolism (FAM) in the progression of AML remains unclear. In this study, we obtained RNA sequencing and corresponding clinicopathological information from the TCGA and GEO databases. Univariate Cox regression analysis and subsequent LASSO Cox regression analysis were utilized to identify prognostic FAM-related genes and develop a potential prognostic risk model. Kaplan-Meier analysis was used for prognostic significances. We also performed ROC curve to illustrate that the risk model in prognostic prediction has good performance. Moreover, significant differences in immune infiltration landscape were found between high-risk and low-risk groups using ESTIMATE and CIBERSOT algorithms. In the end, differential expressed genes (DEGs) were analyzed by gene set enrichment analysis (GSEA) to preliminarily explore the possible signaling pathways related to the prognosis of FAM and AML. The results of our study may provide potential prognostic biomarkers and therapeutic targets for AML patients, which is conducive to individualized precision therapy.

## 1. Introduction

Acute myeloid leukaemia (AML) is a heterogeneous haematological malignancy characterized by abnormal proliferation and differentiation of haemopoietic stem cells (HSCs). AML is highly heterogeneous in its aetiology, pathogenesis, genetic background, clinical presentation, and outcomes [1]. Although many significant breakthroughs in AML immunotherapy and targeted therapy were achieved recently, such as BCL-2 inhibitors, FLT-3 inhibitors, and IDH1/2 inhibitors, postremission relapses occur frequently, and we do not yet have a good understanding of the aetiologies and pathogenesis of AML, which leads to poor prognosis for AML patients [2,3]. Improving the efficacy of AML treatment, especially for patients with relapsed and refractory disease, is still a great challenge. Therefore, further studies on promising prognostic biomarkers and potential therapeutic targets are urgently needed to achieve individualized precision medicine.

Fatty acids, as a crucial part of lipid metabolism, can accumulate to meet the needs of signalling molecules and membranes for lipid synthesis. Growing evidence indicates that fatty acid metabolism is significantly associated with the occurrence and development of multiple cancer types, including AML [4,5,6]. For instance, fatty acid oxidation (FAO) activation was shown to play an essential role in promoting AML cell survival by bone marrow adipocyte remodelling and lipolysis [7]. In addition, upregulating FAO could induce resistance to venetoclax with azacitidine (ven/aza) due to RAS pathway mutations or compensatory adaptations in relapsed disease, so the inhibition of FAO might provide new insights into strategies for ven/aza resistance in AML [8]. Moreover, dysregulation of fatty acids was also demonstrated to affect not only the efficacy of chemotherapy and radiotherapy for cancer patients, but also immunotherapy [9,10]. Moreover, very long-chain fatty acid metabolism also demonstrated a significant connection with the survival of AML cells. For example, very long-chain acyl-CoA dehydrogenase (VLCAD) may cause a decrease in AML cell survival and proliferation by overexpressing and inhibiting fatty FAO in AML cells [11]. In general, the analysis of fatty acid metabolic pathways in AML will contribute to understanding the molecular mechanism of AML and further exploration of novel therapeutic treatments. However, the FAM-related gene set is not thoroughly investigated in AML.

In this study, we developed a reliable and sensitive prognostic risk signature based on 11 FAM-related genes, and thus constructed a nomogram to predict 1-, 3-, and 4-year survival rates. We studied the potential correlation of the risk score with several essential clinical parameters, immune infiltration, and drug sensitivity. The results highlight the essential role of FAM in the AML and provide a novel perspective to understanding metabolic mechanisms.

## 2. Materials and Methods

### 2.1. Data Collection

The gene expression data and corresponding clinical data for 150 AML samples and 337 normal whole-blood samples were retrieved from The Cancer Genome Atlas (TCGA) database and Genotype-Tissue Expression (GTEx) project database, respectively. Study participants with incomplete clinical information were excluded. The transcriptomic and clinical data of 140 patients with AML from the GSE37642 data cohort, which was based on the GPL570 platform, were acquired from the Gene Expression Omnibus (GEO) database and selected as a test set to validate our prognostic signature. Furthermore, a total of 254 fatty acid metabolism-related genes were downloaded from the Molecular Signatures Database (MSigDB) version 7.4 for the next study.

### 2.2. Identification and Enrichment Analysis of the Differentially Expressed Genes (DEGs)

First, Venn analysis was applied to screen overlapping genes between all RNA-seq data (TCGA and GTEx) and FAM-related genes. Then, we selected differentially expressed genes (DEGs) related to FAM from normal and AML samples with the “Limma” R package. A false positive discovery rate (FDR) < 0.05 and|fold change > 0.3|were set as the thresholds. The “Pheatmap” package was applied to make a heatmap t of the DEGs. In addition, gene ontology (GO) analysis and the Kyoto Encyclopedia of Genes and Genomes (KEGG) analysis were conducted to systematically explore the major biological features and cell functional pathways of DEGs using the “org.Hs.e.g.,.db” package and “clusterProfiler” package. Differences with a *p* value < 0.05 were considered statistically significant.

### 2.3. Construction and Validation of the Prognostic Signature Associated with FAM

We used data from the TCGA database and the GEO sample GSE37642 as the training and validation cohort, respectively. The sequencing data of the FAM-related genes of each sample were integrated with corresponding survival data. A univariate Cox regression analysis was conducted to identify key genes associated with the prognosis of AML in the TCGA cohort. Genes with a *p* value < 0.05 were selected for least absolute shrinkage and selection operator (LASSO) analysis to identify optimal prognostic genes. Based on the expression levels and risk coefficients of first-rank prognostic genes, a risk signature was developed, and the risk score for each patient was calculated according to the equation below:

$$\mathrm{Risk}\;\mathrm{score}=\sum_{i=1}^{n}\beta_{i}x_{i}$$

where *n*, $\beta_{i}\;$and$\;x_{i}$ represent the number of signature genes, the value of the correlation coefficients in the LASSO analysis for the genes, and the expression of the gene, respectively. Based on the median risk score, AML patients were separated into high-risk and low-risk categories. Kaplan-Meier analysis and ROC curve analysis were carried out to evaluate the predictive value of the risk signature. We also performed principal component analysis (PCA) to estimate the clustering capacity of the risk signature. In addition, the prognostic performance of the signature was validated in the test set.

### 2.4. Comprehensive Analysis of the Prognostic Risk Score and Clinicopathological Parameters of AML Patients

After excluding missing data in the training group, we combined the risk score of each AML sample with the corresponding clinicopathological characteristics. The connection between the risk score and several clinical characteristics, including FAB subtype, gender, age, cytogenetic, and molecular risk, was investigated by the “limma” R package. Univariate and multivariable Cox regression analyses were performed to determine the independent prognostic factors among the above variables. A *p* < 0.05 was set as the selection criterion.

### 2.5. Development and Assessment of the Nomogram for AML Patients

A novel prognostic nomogram involving all independent prognostic indicators was constructed by the “nomogram” package in R software to further explore individual prognosis. Additionally, calibration, ROC curve and Cox regression analyses were performed to assess the predictive power of the nomogram from different angles.

### 2.6. Gene Set Enrichment Analysis

A total of 216 DEGs were identified between the high-risk and low-risk groups with the screening criteria of FDR < 0.05 and|fold change > 1|. Enrichment analysis of the DEGs was performed with the “Metascape” website. GSEA software was also used to investigate the tumour hallmarks and KEGG pathways in the different subtypes. The pathways and hallmarks with *p* < 0.05 and FDR < 0.25 were regarded as statistically significant.

### 2.7. Immune Infiltration Level Analysis

The ESTIMATE algorithm was applied to estimate the composition of infiltrating stromal and immune cells between different groups. According to the ESTIMATE algorithm, we calculated the immune score and stromal score, which are positively related to the ratio of the corresponding component in the tumour microenvironment (TME), and the ESTIMATE score, which is the integration of the 2 former scores. Then, we quantified the difference in immune cell infiltration and immune function between different groups based on the CIBERSORT algorithm. The associations between the risk score and immune checkpoints were also investigated, and the results are shown in box plots.

### 2.8. Drug Sensitivity Analysis

To explore the potential role of the risk model in the treatment for AML patients, we calculated the IC50 of the antineoplastic drugs using the “pRRophetic” R package [12]. IC50 was an important predictor for evaluating drug response to treatment. All statistical analyses were presented by the R package “ggplot2.”

### 2.9. Protein-Protein Interaction (PPI) Network

The DEGs between the two groups were further explored using STRING online databases, and thus, PPI network data were constructed on the basis of an interaction score > 0.70 (median confidence). We then processed and showed the PPI network data through Cytoscape software, which was used to seek hub genes from all DEGs as a Cytoscape plugin. All samples were divided into low- and high-expression groups according to the median expression value of the hub gene. The expression of the CD163 gene was explored using gene expression profiling interactive analysis (GEPIA) “http://gepia.cancer-pku.cn/index.html (accessed on 7 February 2023), which is an analysis tool that contains RNA sequencing expression data of 9736 tumors and 8587 normal samples from TCGA and the GTEx projects. Furthermore, the relationship of CD163 with the prognosis, clinical features, and immune status were further explored.

### 2.10. RNA Extraction and Real-Time Quantitative PCR (RT-qPCR)

Bone marrow samples for 10 newly diagnosed AML patients and 10 healthy volunteers were collected from the Qilu Hospital of Shandong University in Jinan, China. We used Trizol to extract the total RNA according to specific protocols. Subsequently, the extracted RNA was reversely transcribed into cDNA for qPCR using the Evo M-MLV RT Mix Kit. The 2 × SYBR Green qPCR master mix was applied in RT-qPCR on the Light Cycler 480 II. Relative expressions of the genes were normalized to GAPDH and calculated using the 2^−δδct^ method. The primers used in this study are shown in Table S1.

## 3. Results

**Figure 1:**
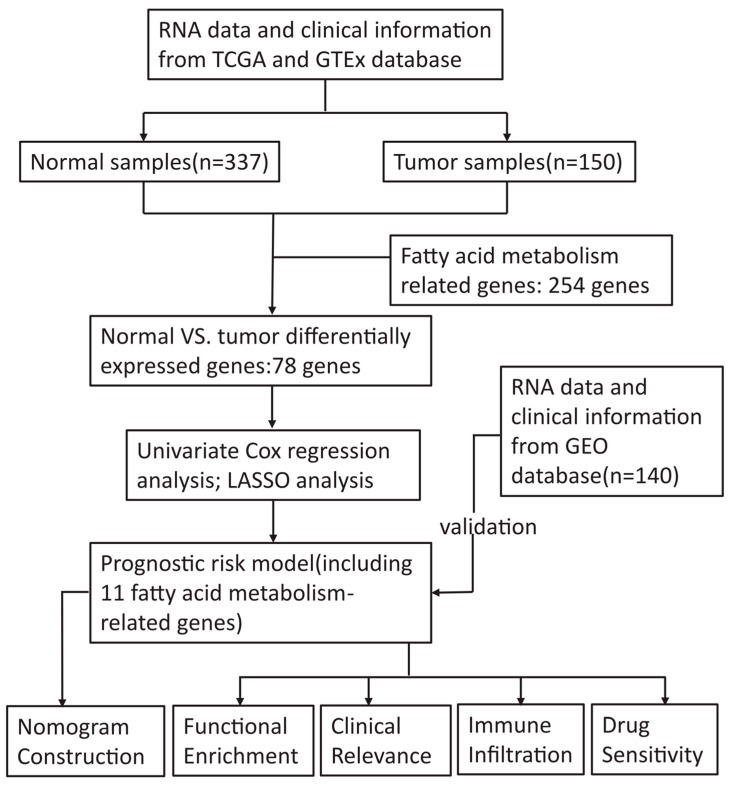
Flow chart of this study.

The detailed flow chart of the study is displayed in Figure 1.

### 3.1. Enrichment Analysis of AML Patient Samples

**Figure 2:**
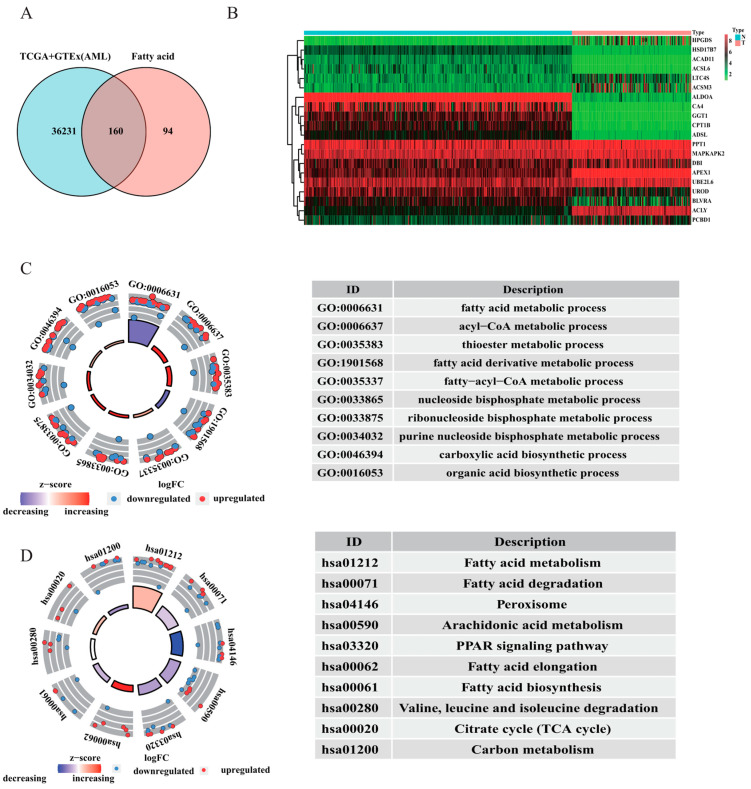
Comparison between AML and normal samples in the TCGA and GTEx databases. (**A**) Venn diagram of FAM-related genes in this study. (**B**) Heatmap of the top 10 upregulated and downregulated FAM-related DEGs in AML and normal samples. (**C**,**D**) GO and KEGG pathway analyses of DEGs related to fatty acid metabolism in AML and normal samples.

As shown in Figure 2A, a total of 160 FAM-related genes were identified. Subsequently, 78 genes were further selected for further study by comparing the expression of FAM-related genes in normal and AML samples. The top 10 upregulated and downregulated genes were visualized on a heatmap (Figure 2B). Enrichment analysis was conducted for these FAM-associated DEGs. Fatty acid metabolism, fatty−acyl−CoA metabolism, and fatty acid derivative metabolism were highlighted in the GO analysis (Figure 2C). The fatty acid metabolism, degradation, biosynthesis, and elongation pathways were significantly clustered in KEGG analysis (Figure 2D). The results indicated that fatty acid metabolism might exert a crucial function in the pathogenesis of AML.

### 3.2. Construction and Validation of the Risk Signature

**Figure 3:**
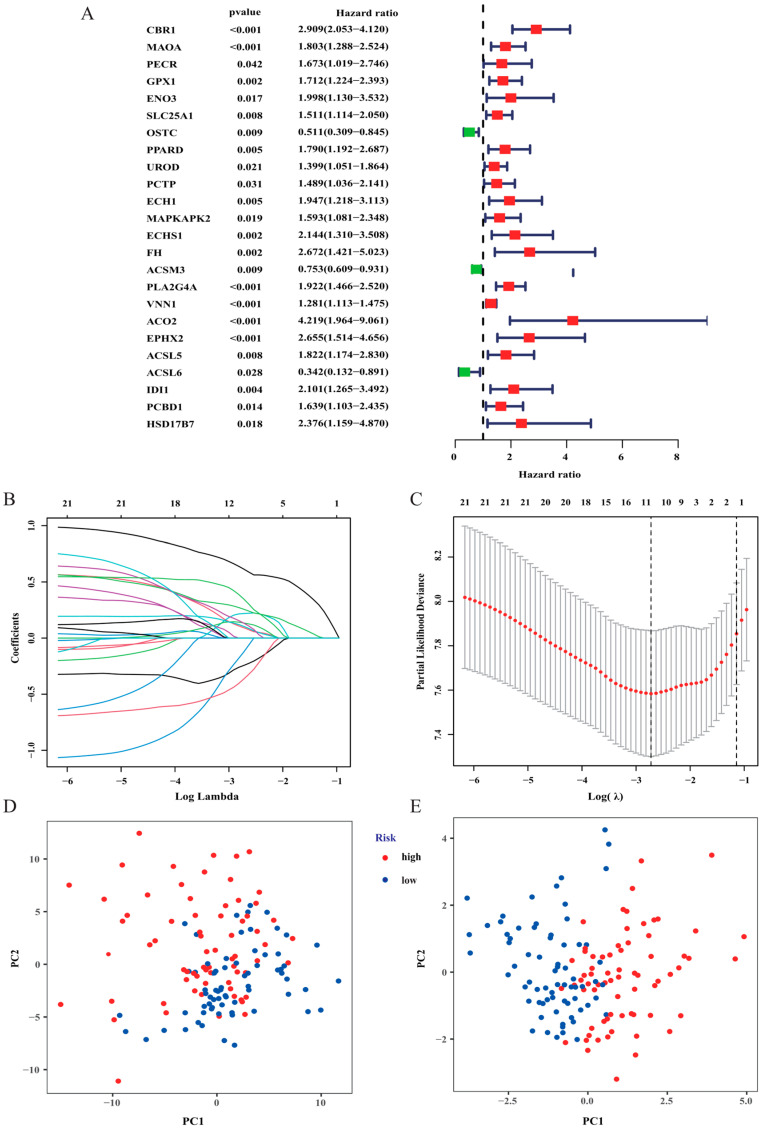
Construction of the prognostic risk model. (**A**) Forest plot of 24 fatty acid metabolism-related genes associated with prognosis identified using univariate Cox regression analysis. (**B**,**C**) LASSO Cox regression was performed to identify fatty acid metabolism-related genes closely associated with the prognosis of AML. (**D**) Principal component analysis based on all fatty acid metabolism-related genes in AML. (**E**) Principal component analysis based on the fatty acid metabolism risk score to distinguish tumours from normal samples. The group marked in blue represents low-risk patients, and the group marked in red represents high-risk patients.

Twenty-four FAM-related genes significantly correlated with the overall survival (OS) of AML patients were identified using universal Cox regression analysis in the TCGA set (Figure 3A). Then, LASSO analysis was applied to further identify the optimal prognostic genes. Finally, 11 FAM-related genes (Table 1) were identified and constituted a prognostic risk model (Figure 3B,C). The risk score of each patient was calculated using the equation: risk score = (0.614 × the expression of the CBR1) + (0.092 × the expression of MAOA) + (0.219 × the expression of ENO3) + (−0.243 × the expression of OSTC) + (0.134 × the expression of UROD) + (−0.123 × the expression of PCTP) + (0.104 × the expression of MAPKAPK2) + (0.257 × the expression of PLA2G4A) + (0.133 × the expression of EPHX2) + (−0.364 × the expression of ACSL6) + (0.348 × the expression of IDI1). The median risk score was applied as a cut-off to categorize AML samples into a high-risk group (*n* = 69) and a low-risk group (*n* = 70). PCA was then utilized to compare the gene expression levels between high- and low-risk patients based on FAM-associated genes and the 11 genes of the prognostic signature (Figure 3D,E). The outcomes revealed that the risk signature had the best discriminatory ability between the two different groups.

**Figure 4:**
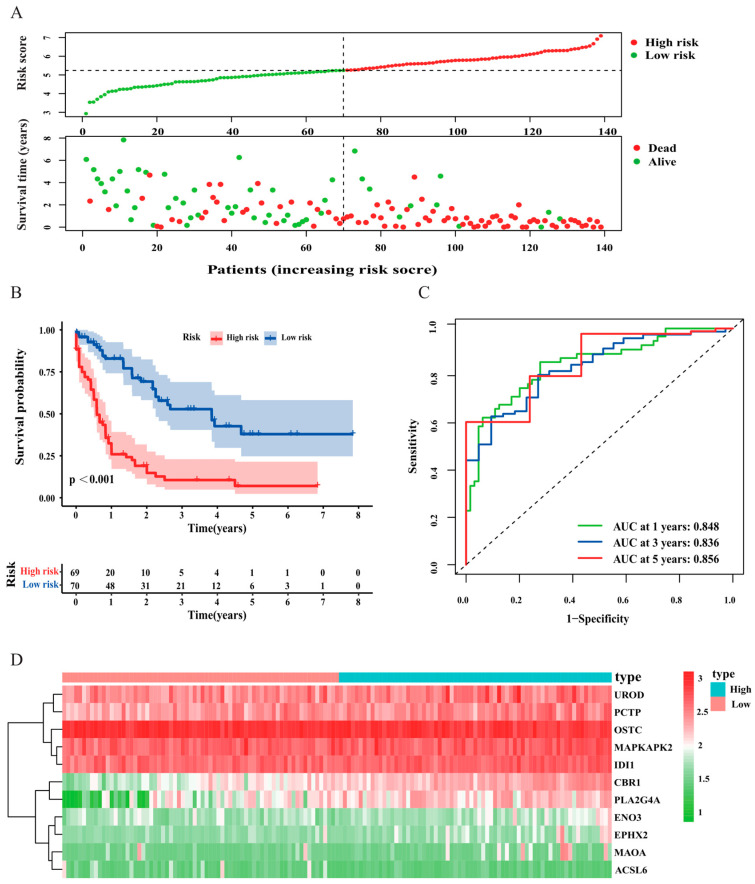
The correlation between risk score and survival status in the training cohort. (**A**) Distribution of the risk scores (**top**) and survival status of each patient (**bottom**). (**B**) Kaplan–Meier curve of the signature. (**C**) ROC curves for predicting survival at 1 year, 3 years, and 5 years. (**D**) Heatmap depicting the differential expression of the 11 genes in AML patients.

The differences in prognosis among AML patients were explored between the different risk groups. The scatter plot displayed that mortality increased with the risk score (Figure 4A). The Kaplan–Meier survival analysis also demonstrated that high risk correlated closely with worse outcome in patients with AML (*p* < 0.001, Figure 4B). Then, we plotted ROC curves of the risk signature to measure predictive sensitivity and specificity, and the areas under the curve (AUCs) for predicting survival at 1, 3, and 5 years were 0.848, 0.836, and 0.856, respectively (Figure 4C). The expression of the 11 prognostic FAM-related genes in the low- and the high-risk group was clearly exhibited in a heatmap diagram (Figure 4D).

**Figure 5:**
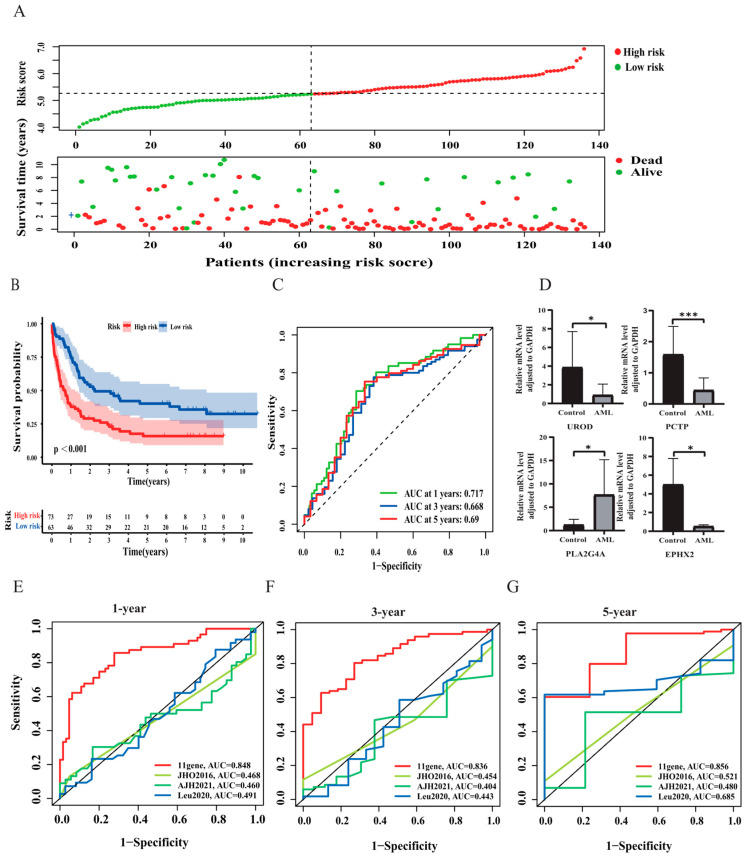
Validation of the risk signature. (**A**,**B**) Risk score distribution and survival status, Kaplan–Meier curve in the GEO cohort. (**C**) ROC curves for predicting survival at 1 year, 3 years, and 5 years in the GEO cohort. (**D**) Validation of the expression of UROD, PCTP, PLA2G4A, and EPHX2 in AML samples by qPCR. (**E**–**G**) AUC comparison between the risk signature based on 11 FAM-related genes and other previously published prognostic signatures. * *p* < 0.05 and *** *p* < 0.001.

To validate the predictive capacity of the risk signature, we used the GEO cohort, which included 140 AML patients, as the test set. GSE37642 was clustered into low- and high-risk groups based on the cut-off value determined in the training set. Consistent with the outcome in the training set, the survival analysis indicated that the risk score had an inverse relationship with the clinical outcomes of AML patients (Figure 5A,B). The AUCs were 0.717, 0.668, and 0.69 for predicting survival at 1, 3, and 5 years, respectively (Figure 5C). We further screened four FAM-related genes from signatures and examined the expressions of these genes by qPCR in 10 AML and 10 healthy control samples. As shown in Figure 5D, the expression levels of UROD, PCTP, and EPHX2 were relatively higher in controls than that in AML, while PLA2G4A were higher in AML. In addition, compared to other known prognostic signatures in AML, such as AJH 2021 [13], Leu 2020 [14], and JHO 2016 [15], the FAM-related risk signature had a significantly better capability to predict prognosis (Figure 5E–G).

Briefly, these results demonstrated that the risk model constructed based on the FAMs prognostic signature could accurately evaluate the prognosis of patients with AML.

### 3.3. Correlation Analysis between Risk Score and Clinicopathological Features

Based on the accuracy of the survival risk predictions, we further investigated the role of the risk signature in predicting AML progression, and the associations between the risk score and clinicopathological characteristics were explored. Significant differences were shown between different groups in age, cytogenetic risk, FAB subtype, and molecular risk (all *p* < 0.001) (Figure S1A–D). However, no correlation was found between the risk score and gender (*p* > 0.05; Figure S1E).

### 3.4. Construction of a Nomogram for AML Patients

**Figure 6:**
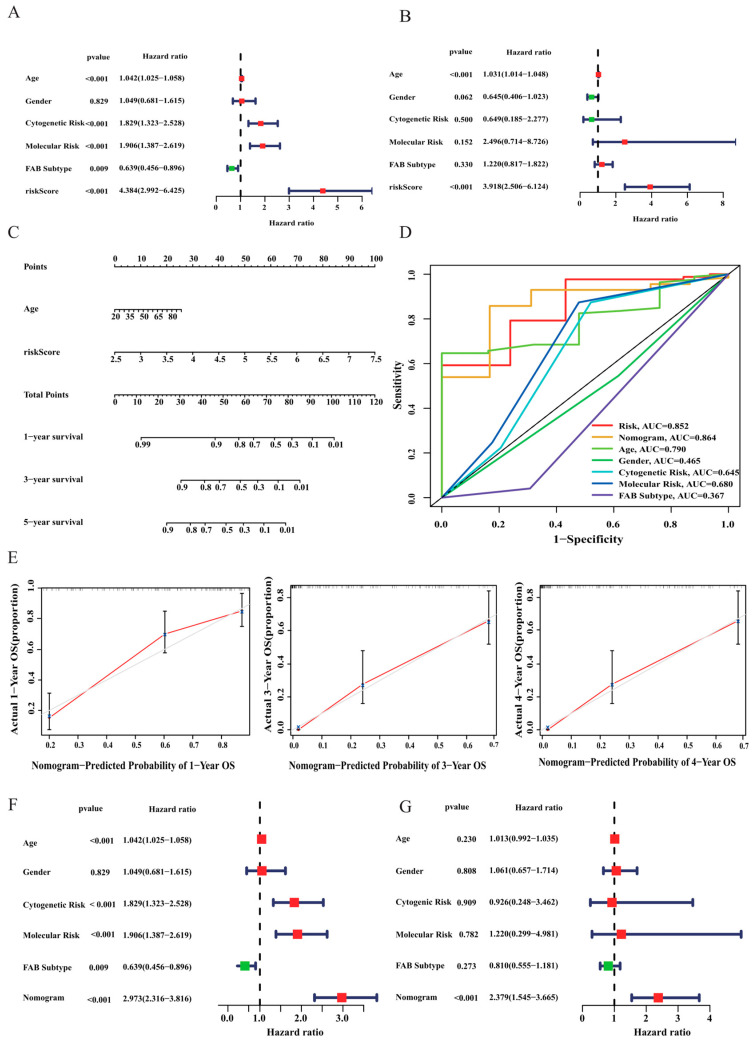
Development and assessment of the nomogram for patients with AML. (**A**,**B**) Univariate and multivariate Cox regression analyses of clinical parameters in patients with AML. (**C**) Nomogram to predict the 1-, 3-, and 4-year OS of AML patients. (**D**) ROC curves of the nomogram, risk score, and clinical characteristics in predicting prognosis. (**E**) Calibration plot analysis to evaluate the predictive ability of the nomogram. The *x*-axis is nomogram-predicted survival, and the *y*-axis is actual survival. (**F**,**G**) Univariate and multivariate Cox regression analyses were conducted to identify if the nomogram score was an independent predictor in AML patients. Green squares: Hazard ratio (HR) < 1; red squares: HR > 1.

To identify the independent predictors of survival in AML patients, univariate and multivariate Cox regression analyses were performed. The results indicate that risk score and age were the only two independent prognostic factors for predicting the OS of AML patients (*p* < 0.001; Figure 6A,B). Subsequently, a prognostic nomogram including independent risk factors (age and risk score) was constructed to predict the survival probabilities of AML patients at 1, 3, and 4 years (Figure 6C). The AUCs showed that the nomogram (AUC = 0.864) had higher sensitivity and specificity than other single prognostic factors, such as age (AUC = 0.790), cytogenetic risk (AUC = 0.645), molecular risk (AUC = 0.680), and risk score (AUC = 0.852) (Figure 6D). In addition, the calibration curves demonstrated good prediction performance, and our model was similar to the ideal model in estimating 1-, 3-, and 4-year OS (Figure 6E). Moreover, Cox regression analyses were conducted to confirm that the nomogram score was an independent predictive factor for AML prognosis in all participants (Figure 6F,G). Collectively, we validated the prominently predictive ability of the tumour prognostic signature and revealed a high potential for clinical utility from multiple perspectives.

### 3.5. Functional and Annotation Analyses

**Figure 7:**
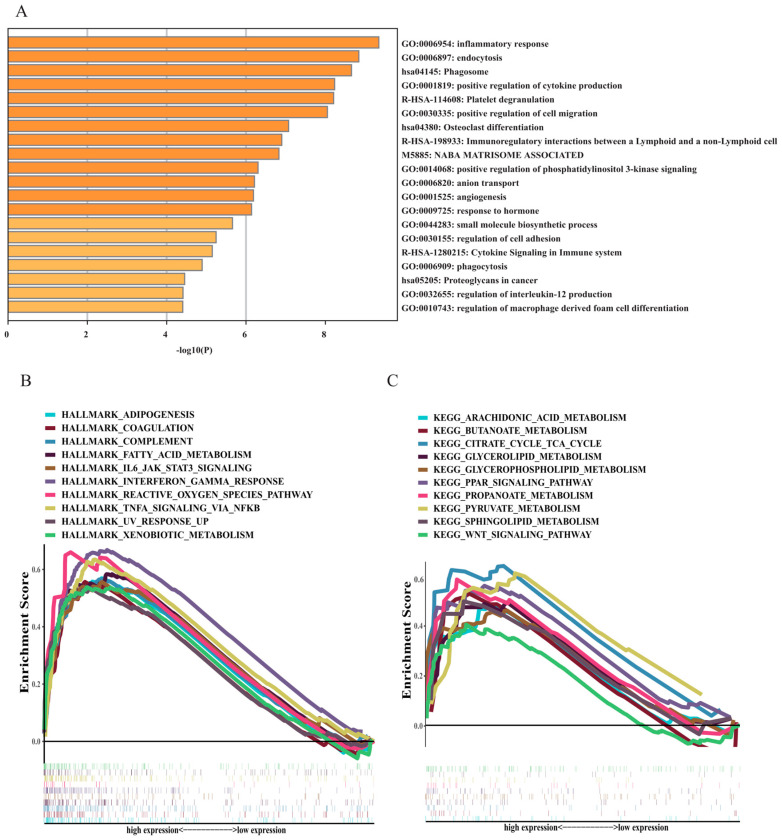
Function and pathway enrichment analysis. (**A**) Function and pathway enrichment analysis by Metascape. The image shows the histogram of the top 20 enriched pathways. (**B**,**C**) GSEA results.

To distinguish the biological functions and networks related to the risk signature, we screened 216 DEGs in the low- and high-risk group for enrichment analyses. Metascape analysis results establish that some immune-associated pathways, including cytokine signalling in the immune system, regulation of interleukin-12 production, regulation of macrophage-derived foam cell differentiation, and several pathways associated with the development of malignant tumours, such as positive regulation of phosphatidylinositol 3-kinase signalling and proteoglycans [16,17] in cancer, were significantly clustered (Figure 7A). Moreover, GSEA was also conducted for functional enrichment analysis. The results using the Hallmark database are shown in Figure 7B. Most metabolism-related pathways, such as fatty acid metabolism and adipogenesis, and several immune pathways, such as TNF-α signalling via NF-κB pathways and IL6-JAK-STAT3 signalling, were significantly enriched in the high-risk group. Additionally, the results using the KEGG database demonstrate that fatty acid substance metabolism pathways, such as arachidonic acid and butanoate acid metabolism, glycerolipid metabolism, propanoate metabolism, pyruvate metabolism, and associated fatty acid metabolism pathways, such as citrate cycle tac cycle signalling pathways (TCA cycle) and PPAR signalling pathways, were greatly clustered in the high-risk group (Figure 7C). All these results validated that FAM-related signatures are closely connected to the immune response, which is crucial for AML.

### 3.6. The Landscape of the Tumour Microenvironment (TME) and Immune Cell Infiltration in AML Patients

**Figure 8:**
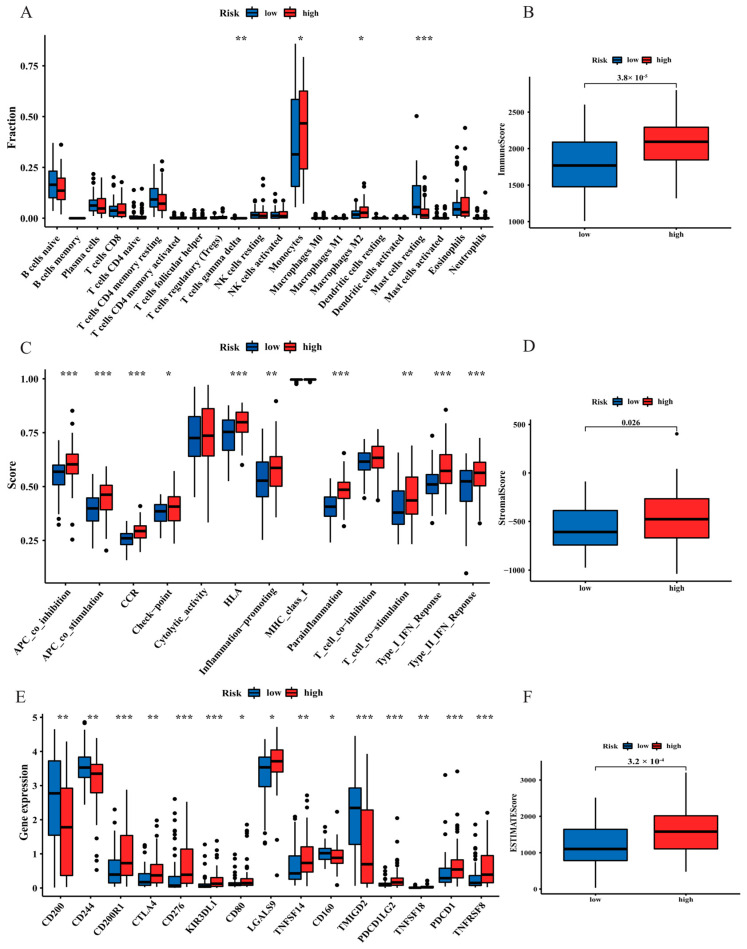
Comparison of the tumour microenvironment (TME) and immune cell infiltration between the two risk groups. (**A**) Box plot of the fraction of 22 immune cells in the high- and low-risk group. (**B**,**D**,**F**) Immune score, stromal score, and ESTIMATE score between different risk groups. (**C**) Box plot of the scores of 13 immune-related functions in the high- and low-risk group. (**E**) Differences in the expression of 15 checkpoints in the two risk groups. * *p* < 0.05; ** *p* < 0.01; and *** *p* < 0.001.

According to the enrichment analysis, pathways related to fatty acid metabolism and immunity were significantly highlighted in the high-risk group, suggesting that exploration of the correlation between immune status and risk score was essential. The result from CIBERSORT algorithm revealed that monocytes and macrophage M2 cells infiltrated at a greater rate in the high-risk group, while resting mast cells were markedly activated in the low-risk group (*p* < 0.05; Figure 8A). Higher immune, stromal, and ESTIMATE scores were found in the patients with the high risk using the ESTIMATE algorithm (*p* < 0.05, Figure 8B,D,F), representing a relatively high infiltration of immune and stromal cells in the high-risk TME, which was associated with worse prognosis [18,19]. Regarding immune-associated functions, the type I IFN and II response, APC co-inhibition and stimulation, checkpoint, chemokine receptors (CCRs), inflammation promotion, HLA, T cell co-stimulation, and parainflammation were better activated in the high-risk group (*p* < 0.05, Figure 8C). Moreover, there were drastic differences in immune checkpoint expression. The expression levels of immune checkpoints, such as CTLA4 and PDCD1, were positively correlated with the risk score (*p* < 0.05, Figure 8E).

### 3.7. Analysis of Drug Sensitivity in the Two Risk Groups

**Figure 9:**
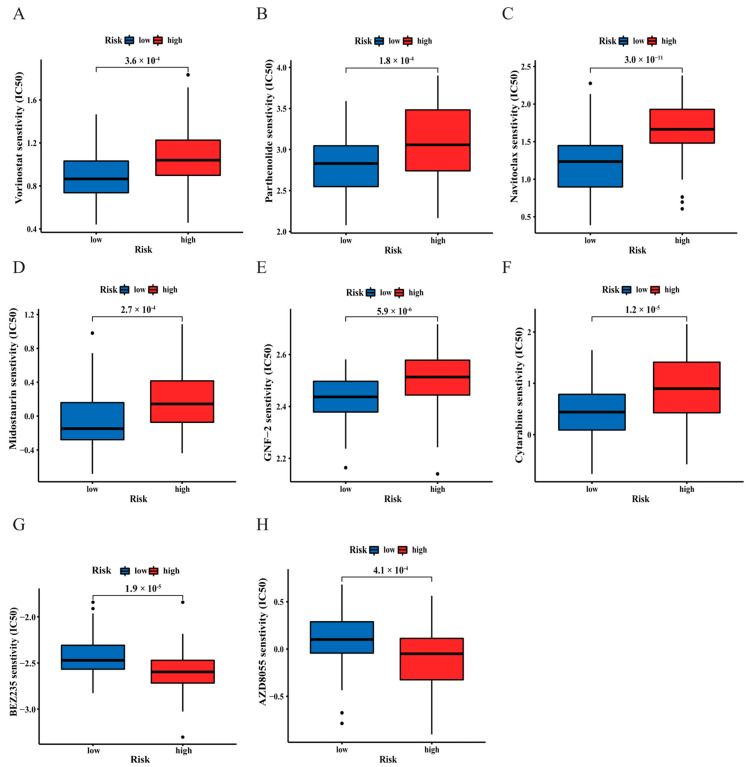
Drug sensitivity predictions in different risk groups. (**A**–**H**) Vorinostat, parthenolide, navitoclax, midostaurin, GNF-2, cytarabine (Ara-C), BEZ235, and AZD8055.

Chemotherapy and targeted drug therapy are considered as vital strategies in the clinical management of AML patients. Therefore, it is necessary to explore differences in drug sensitivity between the different risk groups. In our study, the traditional cytotoxic drugs were more sensitive in the low-risk group and included histone deacetylase (HDAC) inhibitors, such as vorinostat and parthenolide (Figure 9A,B), the BCL-2 inhibitor navitoclax (Figure 9C), and midostaurin (Figure 9D), which were demonstrated to consolidate chemotherapy and encourage efficacy in AML patients with a FLT3 mutation [20]. GNF-2, as an allosteric inhibitor of BCR-ABL, also showed higher sensitivity in the low-risk group (Figure 9E). Moreover, a more sensitive response to cytarabine (Ara-C) was seen in the low-risk group than in the high-risk group (Figure 9F). Ara-C is a first-line agent with excellent activity in AML. However, the PI3K/mTOR inhibitors BEZ235 and AZD8055 were less sensitive in the low-risk group (Figure 9G,H). According to above results, the two risk groups responded dramatically different to chemotherapy.

### 3.8. PPI Analysis 

**Figure 10:**
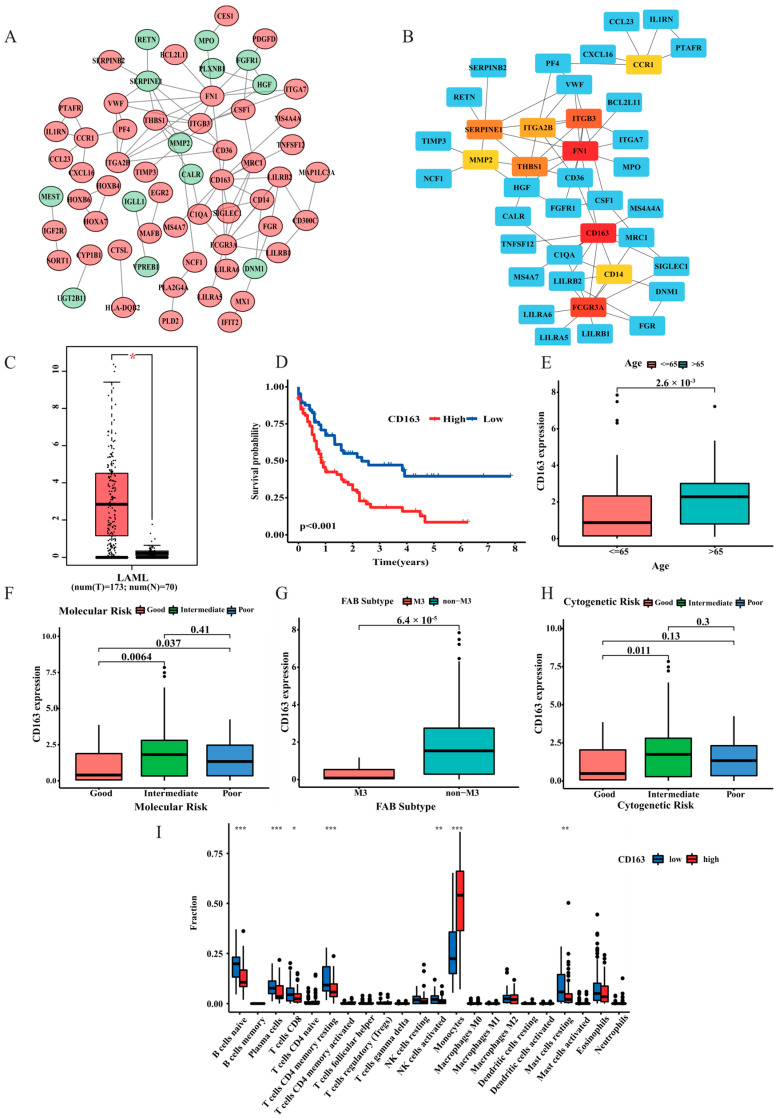
Protein-protein interaction (PPI) network. (**A**) PPI network processed by Cytoscape. Upregulated genes in the high-risk score group are marked in red, and those in the low-risk score group are presented in green. (**B**) Top 10 hub genes selected by cytoHubba. (**C**) The expression of CD163 genes in AML patients and paired normal bone marrow samples was analyzed by GEPIA. (**D**) Survival analysis for the subgroup classified by CD163 mRNA expression. (**E**–**H**) The CD163 mRNA expression levels differed among different groups based on clinical features, including age, molecular risk, FAB subtype, and cytogenetic risk. (**I**) Immune cell infiltration in patients with different expression levels of CD163. The text continues here. * *p* < 0.05; ** *p* < 0.01; and *** *p* < 0.001.

To further investigate the discrepancies between the low- and high-risk group, the DEGs of the two risk groups were introduced into the online database STRING to analyze their expression profiles. Relevant PPIs were obtained and are visualized in Figure S2A. As shown in Figure 10A, the interacting genes were processed using Cytoscape software (version 3.9.0), which is an open source software platform for visualizing complex networks and integrating these with any type of attribute data. Cytoscape and the plug-in app “cytoHubba” were applied to parse the network. The ten highest-scored genes (including CD163, FN1, FCGR3A, ITGB3, SERPINE1, THBS1, ITGA2B, CD14, MMP2, and CCR1) in the network were identified as the hub genes on the basis of the “MCC” algorithm, which are shown in Figure 10B. Subsequently, we analyzed CD163 expression level in AML patients by GEPIA dataset. Result show CD163 mRNA expression was significantly higher in AML tissues compared to that in the corresponding healthy bone marrow samples (Figure 10C). Kaplan-Meier analysis was also performed to determine whether hub gene expression correlated with AML survival. Except for FN1 and FCGR3A, the other eight hub genes were significantly related to the prognoses of AML patients (*p* < 0.05, Figure S2B–J; Figure 10D). Among all hub genes, CD163 was observed to be the most relevant prognostic factor. In addition, the selection of the core genes in the PPI network also indicated that CD163 was centrally located. These results suggest that CD163 may serve as a novel therapeutic target in AML. Therefore, we divided all AML samples into two groups according to the CD163 mRNA median expression value. The correlation study between CD163 expression and AML clinical status revealed that age, cytogenetic risk, molecular risk, and FAB subtype were closely related to CD163 expression (Figure 10E–H), suggesting an inverse correlation between CD163 expression and the clinical status and prognosis of AML. In addition, we also conducted an investigation to explore the specific differences in immune cell infiltration between groups with high and low CD163 expression. The results show that NK, B cell, and CD8+ T cell infiltration was significantly increased in tumours with low CD163 expression (Figure 10I). 

## 4. Discussion

AML is an extremely heterogeneous disease due to its complicated genetic and molecular mechanisms. Despite excellent breakthroughs in targeted therapy, the complete remission (CR) rates and long-term survival of AML patients are still unsatisfactory [21]. Therefore, the identification of effective prognostic biomarkers is urgently needed. FAM is involved in membrane synthesis, energy generation, and signal transduction in AML, including tumorigenesis and the progression of cancer [22]. Although recent literature demonstrated that multiple FAM-related genes could greatly improve prognostic prediction compared with a single biomarker in AML [23], we further investigated the correlation between FAM-related genes and the OS of AML patients, and thus selected 11 new genes to construct a risk model for prognostic prediction and therapeutic decision guidance. Moreover, we provided a hybrid nomogram model combining clinical factors with an effective quantitative scoring method that could predict the OS of AML patients.

Among these 11 new genes in the prognostic signature, carbonyl reductase 1 (CBR1) was illustrated to have an essential role in the metabolism of multiple drugs, such as daunorubicin, doxorubicin, anthracycline, and haloperidol [24,25]. Daunorubicin, as a great choice for patients with haematologic malignancies, including AML, was demonstrated to be affected by CBR1 [26]. Phospholipase A2-IVA (PLA2G4A) not only plays a vital role in the development of various solid tumours [27,28], but was also illustrated to independently predict OS in patients with non-M3/NPM1 WT AML [29]. ACSL6, an isoform of the acyl-CoA synthetase long-chain family (ACSL), can promote leukaemogenesis by ASL6-ETV6 fusion gene formation [30]. MAPKAPK2 [31], MAOA [32], and EPHX2 [33] were demonstrated to affect tumour development in various contexts. ENO3, OSTC, and UROD serve as diagnostic or prognostic biomarkers in other cancers [34,35,36]. To date, the function of PCTP and IDI1 in the progression of cancers is still unclear, and further experimental work is needed. Generally, the above evidence suggests that these 11 newly identified genes have the potential to become novel markers for the prediction of prognosis and to become targets for molecular targeted therapy in patients with AML.

Functional and annotation analyses indicated that FAM-associated pathways and immune-related hallmarks were markedly highlighted in the high-risk group, suggesting that the level of fatty acids may affect the function and phenotype of infiltrating immune cells in the microenvironment [37]. Immune regulation plays an essential role in the initiation and development of AML. The number and proportion of infiltrating immune cells can impact cancer progression, prognosis prediction, and immunotherapy response [38]. According to the immune cell infiltration results, monocyte and macrophage M2 cells were highly expressed in the high-risk group, which are signs of immune evasion. The polarization of macrophages towards the M2 phenotype is favoured for the development of tumour cells, angiogenesis, and immunosuppression. In addition, a higher number of M2-like macrophages in the TME is associated with poorer outcomes in several malignant tumours, including AML [39,40]. This evidence indicates that the poor prognosis of high-risk patients is positively associated with immunosuppression in the TME, and these differences would favour tumour progression and immunotherapy response.

In view of the significant discrepancies observed between the low-risk and high-risk groups, further investigations of the different genes in each group were needed. It was discovered that CD163 is crucial. As a haemoglobin scavenger receptor, the overexpression of CD163 was observed to be significantly correlated with tumour progression and poor prognosis in multiple human cancers [41], which is consistent with our results. In addition, CD163 was identified as a vital biomarker of M2 macrophage activation and may predict the invasion and prognosis of malignancies [42,43]. Therefore, CD163 might provide a potential therapeutic target for the treatment of haematologic malignancies.

Overall, this study meticulously constructed and verified a novel prognostic signature based on FAM-associated genes for patients with AML. A comprehensive investigation of the associations of signalling pathways, drug sensitivity, and immune infiltration with the prognostic risk model for leukaemia was conducted, and these findings may provide a novel basis for more precise targeted therapies in AML. However, a few shortcomings and drawbacks of our study should be taken into consideration. The results based on bioinformatics in this paper were not verified by in vitro experiments. In the future, we will experimentally verify the significance of fatty acid metabolism in AML.

## 5. Conclusions

A novel fatty acid metabolism-associated risk model was successfully constructed and demonstrated a reliable prognostic ability for chemotherapy sensitivity and response to future antitumour immunotherapy. In addition, we generated a nomogram to accurately predict the 1-, 3-, and 4-year survival rates using only several variables from AML patients.

## Figures and Tables

**Table 1:** Eleven fatty acid metabolism-related genes detected using LASSO regression analysis.

Gene	Coefficient
CBR1	0.614533407779894
MAOA	0.0923271597975086
ENO3	0.21850701264524
OSTC	−0.243479390920753
UROD	0.134439432274683
PCTP	−0.123309988656909
MAPKAPK2	0.104413688378819
PLA2G4A	0.25685461709233
EPHX2	0.133099892317426
ACSL6	−0.364168757158391
IDI1	0.347710847872942

## Data Availability

The data used to support the findings of this study are available from the corresponding authors upon request.

## Supplementary Materials

The following supporting information can be downloaded at: https://www.mdpi.com/article/10.3390/curroncol30020193/s1, Figure S1: Correlation between the risk score and clinicopathological characteristics in the TCGA cohort. Figure S2: Construction of the PPI network and prognostic value of the top 10 hub genes. Table S1: The primer sequences of four FAM-related genes.

## References

[1] Heuser M., Ofran Y., Boissel N., Brunet Mauri S., Craddock C., Janssen J., Wierzbowska A., Buske C., ESMO Guidelines Committee (2020). Acute myeloid leukaemia in adult patients: ESMO Clinical Practice Guidelines for diagnosis, treatment and follow-up. Ann. Oncol..

[2] Guieze R., Liu V.M., Rosebrock D., Jourdain A.A., Hernandez-Sanchez M., Martinez Zurita A., Sun J., Ten Hacken E., Baranowski K., Thompson P.A. (2019). Mitochondrial Reprogramming Underlies Resistance to BCL-2 Inhibition in Lymphoid Malignancies. Cancer Cell.

[3] Tallman M.S., Wang E.S., Altman J.K., Appelbaum F.R., Bhatt V.R., Bixby D., Coutre S.E., De Lima M., Fathi A.T., Fiorella M. (2019). Acute Myeloid Leukemia, Version 3.2019, NCCN Clinical Practice Guidelines in Oncology. J. Natl. Compr. Canc. Netw..

[4] Madak-Erdogan Z., Band S., Zhao Y.C., Smith B.P., Kulkoyluoglu-Cotul E., Zuo Q., Santaliz Casiano A., Wrobel K., Rossi G., Smith R.L. (2019). Free Fatty Acids Rewire Cancer Metabolism in Obesity-Associated Breast Cancer via Estrogen Receptor and mTOR Signaling. Cancer Res..

[5] He D., Cai L., Huang W., Weng Q., Lin X., You M., Liao S. (2021). Prognostic value of fatty acid metabolism-related genes in patients with hepatocellular carcinoma. Aging.

[6] Wang Y.N., Zeng Z.L., Lu J., Wang Y., Liu Z.X., He M.M., Zhao Q., Wang Z.X., Li T., Lu Y.X. (2018). CPT1A-mediated fatty acid oxidation promotes colorectal cancer cell metastasis by inhibiting anoikis. Oncogene.

[7] Tabe Y., Konopleva M., Andreeff M. (2020). Fatty Acid Metabolism, Bone Marrow Adipocytes, and AML. Front. Oncol..

[8] Stevens B.M., Jones C.L., Pollyea D.A., Culp-Hill R., D’Alessandro A., Winters A., Krug A., Abbott D., Goosman M., Pei S. (2020). Fatty acid metabolism underlies venetoclax resistance in acute myeloid leukemia stem cells. Nat. Cancer.

[9] Han S., Wei R., Zhang X., Jiang N., Fan M., Huang J.H., Xie B., Zhang L., Miao W., Butler A.C. (2019). CPT1A/2-Mediated FAO Enhancement-A Metabolic Target in Radioresistant Breast Cancer. Front. Oncol..

[10] Wu Y., Fabritius M., Ip C. (2009). Chemotherapeutic sensitization by endoplasmic reticulum stress: Increasing the efficacy of taxane against prostate cancer. Cancer Biol. Ther..

[11] Tcheng M., Roma A., Ahmed N., Smith R.W., Jayanth P., Minden M.D., Schimmer A.D., Hess D.A., Hope K., Rea K.A. (2021). Very long chain fatty acid metabolism is required in acute myeloid leukemia. Blood.

[12] Geeleher P., Cox N., Huang R.S. (2014). pRRophetic: An R package for prediction of clinical chemotherapeutic response from tumor gene expression levels. PLoS ONE.

[13] Chen Z., Song J., Wang W., Bai J., Zhang Y., Shi J., Bai J., Zhou Y. (2021). A novel 4-mRNA signature predicts the overall survival in acute myeloid leukemia. Am. J. Hematol..

[14] Elsayed A.H., Rafiee R., Cao X., Raimondi S., Downing J.R., Ribeiro R., Fan Y., Gruber T.A., Baker S., Klco J. (2020). A six-gene leukemic stem cell score identifies high risk pediatric acute myeloid leukemia. Leukemia.

[15] Wilop S., Chou W.C., Jost E., Crysandt M., Panse J., Chuang M.K., Brummendorf T.H., Wagner W., Tien H.F., Kharabi Masouleh B. (2016). A three-gene expression-based risk score can refine the European LeukemiaNet AML classification. J. Hematol. Oncol..

[16] Nepstad I., Hatfield K.J., Gronningsaeter I.S., Reikvam H. (2020). The PI3K-Akt-mTOR Signaling Pathway in Human Acute Myeloid Leukemia (AML) Cells. Int. J. Mol. Sci..

[17] Espinoza-Sanchez N.A., Gotte M. (2020). Role of cell surface proteoglycans in cancer immunotherapy. Semin. Cancer Biol..

[18] Deng X., Lin D., Zhang X., Shen X., Yang Z., Yang L., Lu X., Yu L., Zhang N., Lin J. (2020). Profiles of immune-related genes and immune cell infiltration in the tumor microenvironment of diffuse lower-grade gliomas. J. Cell. Physiol..

[19] Jiang X., Yan Q., Xie L., Xu S., Jiang K., Huang J., Wen Y., Yan Y., Zheng J., Tang S. (2021). Construction and Validation of a Ferroptosis-Related Prognostic Model for Gastric Cancer. J. Oncol..

[20] Stone R.M., Mandrekar S.J., Sanford B.L., Laumann K., Geyer S., Bloomfield C.D., Thiede C., Prior T.W., Dohner K., Marcucci G. (2017). Midostaurin plus Chemotherapy for Acute Myeloid Leukemia with a FLT3 Mutation. N. Engl. J. Med..

[21] Short N.J., Rytting M.E., Cortes J.E. (2018). Acute myeloid leukaemia. Lancet.

[22] Currie E., Schulze A., Zechner R., Walther T.C., Farese R.V. (2013). Cellular fatty acid metabolism and cancer. Cell Metab..

[23] Zhang H.B., Sun Z.K., Zhong F.M., Yao F.Y., Liu J., Zhang J., Zhang N., Lin J., Li S.Q., Li M.Y. (2022). A novel fatty acid metabolism-related signature identifies features of the tumor microenvironment and predicts clinical outcome in acute myeloid leukemia. Lipids Health Dis..

[24] Plebuch M., Soldan M., Hungerer C., Koch L., Maser E. (2007). Increased resistance of tumor cells to daunorubicin after transfection of cDNAs coding for anthracycline inactivating enzymes. Cancer Lett..

[25] Olson L.E., Bedja D., Alvey S.J., Cardounel A.J., Gabrielson K.L., Reeves R.H. (2003). Protection from doxorubicin-induced cardiac toxicity in mice with a null allele of carbonyl reductase 1. Cancer Res..

[26] Varatharajan S., Abraham A., Zhang W., Shaji R.V., Ahmed R., Abraham A., George B., Srivastava A., Chandy M., Mathews V. (2012). Carbonyl reductase 1 expression influences daunorubicin metabolism in acute myeloid leukemia. Eur. J. Clin. Pharmacol..

[27] Zhang W., Wang X., Zhang L., Geng D., Wang Y., Sun D., Sui P., Zhao X., Xin C., Jiang J. (2018). Inhibition of PLA2G4A Reduces the Expression of Lung Cancer-Related Cytokines. DNA Cell Biol..

[28] Zhan Y., Zheng L., Liu J., Hu D., Wang J., Liu K., Guo J., Zhang T., Kong D. (2021). PLA2G4A promotes right-sided colorectal cancer progression by inducing CD39+gammadelta Treg polarization. JCI Insight.

[29] Bai H., Zhou M., Zeng M., Han L. (2020). PLA2G4A Is a Potential Biomarker Predicting Shorter Overall Survival in Patients with Non-M3/NPM1 Wildtype Acute Myeloid Leukemia. DNA Cell Biol..

[30] Baldazzi C., Luatti S., Marzocchi G., Grassi A., Cavo M., Testoni N. (2022). t(5;12)(q31;p13)/ETV6::ACSL6 and t(6;9)(p23;q34)/DEK::NUP214 concurrence in acute myeloid leukemia: An unusual association of two rare abnormalities. Cancer Genet..

[31] Soni S., Anand P., Padwad Y.S. (2019). MAPKAPK2: The master regulator of RNA-binding proteins modulates transcript stability and tumor progression. J. Exp. Clin. Cancer Res..

[32] Li J., Pu T., Yin L., Li Q., Liao C.P., Wu B.J. (2020). MAOA-mediated reprogramming of stromal fibroblasts promotes prostate tumorigenesis and cancer stemness. Oncogene.

[33] Zhan K., Bai Y., Liao S., Chen H., Kuang L., Luo Q., Lv L., Qiu L., Mei Z. (2021). Identification and validation of EPHX2 as a prognostic biomarker in hepatocellular carcinoma. Mol. Med. Rep..

[34] Berk L.S., Webb G., Imperio N.C., Nehlsen-Cannarella S.L., Eby W.C. (1986). Simple, rapid 125I-labeled cyclosporine double antibody/polyethylene glycol radioimmunoassay used in a pediatric cardiac transplant program. Ther. Drug Monit..

[35] Zhang J., Lu H., Zhang S., Wang T., Zhao H., Guan F., Zeng P. (2021). Leveraging Methylation Alterations to Discover Potential Causal Genes Associated With the Survival Risk of Cervical Cancer in TCGA Through a Two-Stage Inference Approach. Front. Genet..

[36] Ito E., Yue S., Moriyama E.H., Hui A.B., Kim I., Shi W., Alajez N.M., Bhogal N., Li G., Datti A. (2011). Uroporphyrinogen decarboxylase is a radiosensitizing target for head and neck cancer. Sci. Transl. Med..

[37] Lochner M., Berod L., Sparwasser T. (2015). Fatty acid metabolism in the regulation of T cell function. Trends Immunol..

[38] Yao J., Chen X., Liu X., Li R., Zhou X., Qu Y. (2021). Characterization of a ferroptosis and iron-metabolism related lncRNA signature in lung adenocarcinoma. Cancer Cell Int..

[39] Liu J.Y., Peng C.W., Yang G.F., Hu W.Q., Yang X.J., Huang C.Q., Xiong B., Li Y. (2017). Distribution pattern of tumor associated macrophages predicts the prognosis of gastric cancer. Oncotarget.

[40] Xu Z.J., Gu Y., Wang C.Z., Jin Y., Wen X.M., Ma J.C., Tang L.J., Mao Z.W., Qian J., Lin J. (2020). The M2 macrophage marker CD206: A novel prognostic indicator for acute myeloid leukemia. Oncoimmunology.

[41] Yan H., Qu J., Cao W., Liu Y., Zheng G., Zhang E., Cai Z. (2019). Identification of prognostic genes in the acute myeloid leukemia immune microenvironment based on TCGA data analysis. Cancer Immunol. Immunother..

[42] Stilund M., Gjelstrup M.C., Petersen T., Moller H.J., Rasmussen P.V., Christensen T. (2015). Biomarkers of inflammation and axonal degeneration/damage in patients with newly diagnosed multiple sclerosis: Contributions of the soluble CD163 CSF/serum ratio to a biomarker panel. PLoS ONE.

[43] Hu J.M., Liu K., Liu J.H., Jiang X.L., Wang X.L., Chen Y.Z., Li S.G., Zou H., Pang L.J., Liu C.X. (2017). CD163 as a marker of M2 macrophage, contribute to predicte aggressiveness and prognosis of Kazakh esophageal squamous cell carcinoma. Oncotarget.

