# Supplementary material for: A Novel Fatty Acid Metabolism-Associated Risk Model for Prognosis Prediction in Acute Myeloid Leukaemia
Source: Curr Oncol. 2023 Feb 20;30(2):2524–42. doi: 10.3390/curroncol30020193 (PMC9955245; doi:10.3390/curroncol30020193)
Supplement: Supplementary file 1 [file curroncol-30-00193-s001.zip › curroncol-2131219-supplementary.pdf]

Current Oncology

MDPI

Supplementary Materials

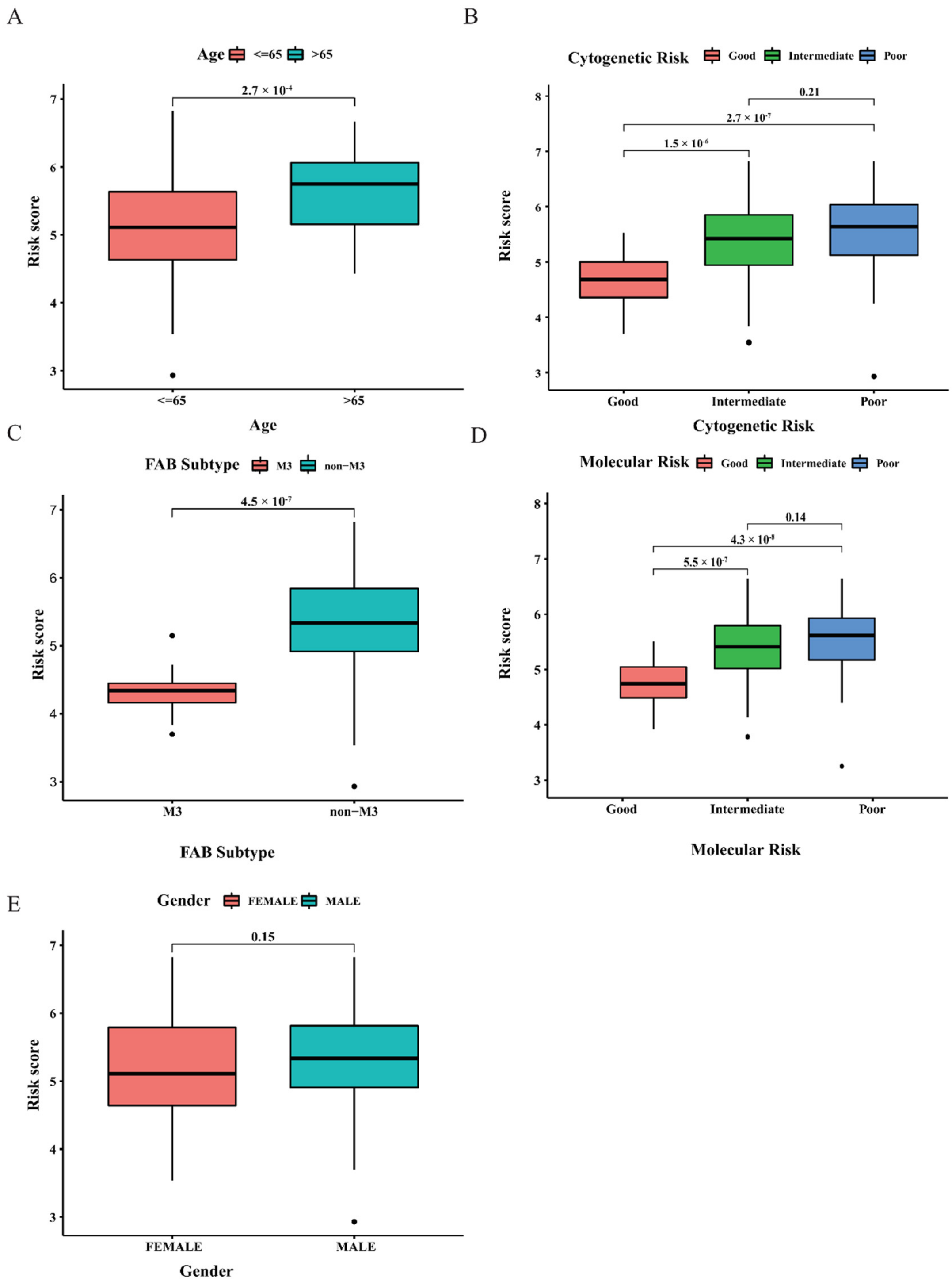

Figure S1. Correlation between the risk score and clinicopathological characteristics in the TCGA cohort. (**A-E**) Age, cytogenetic risk, FAB subtype, molecular risk and gender.

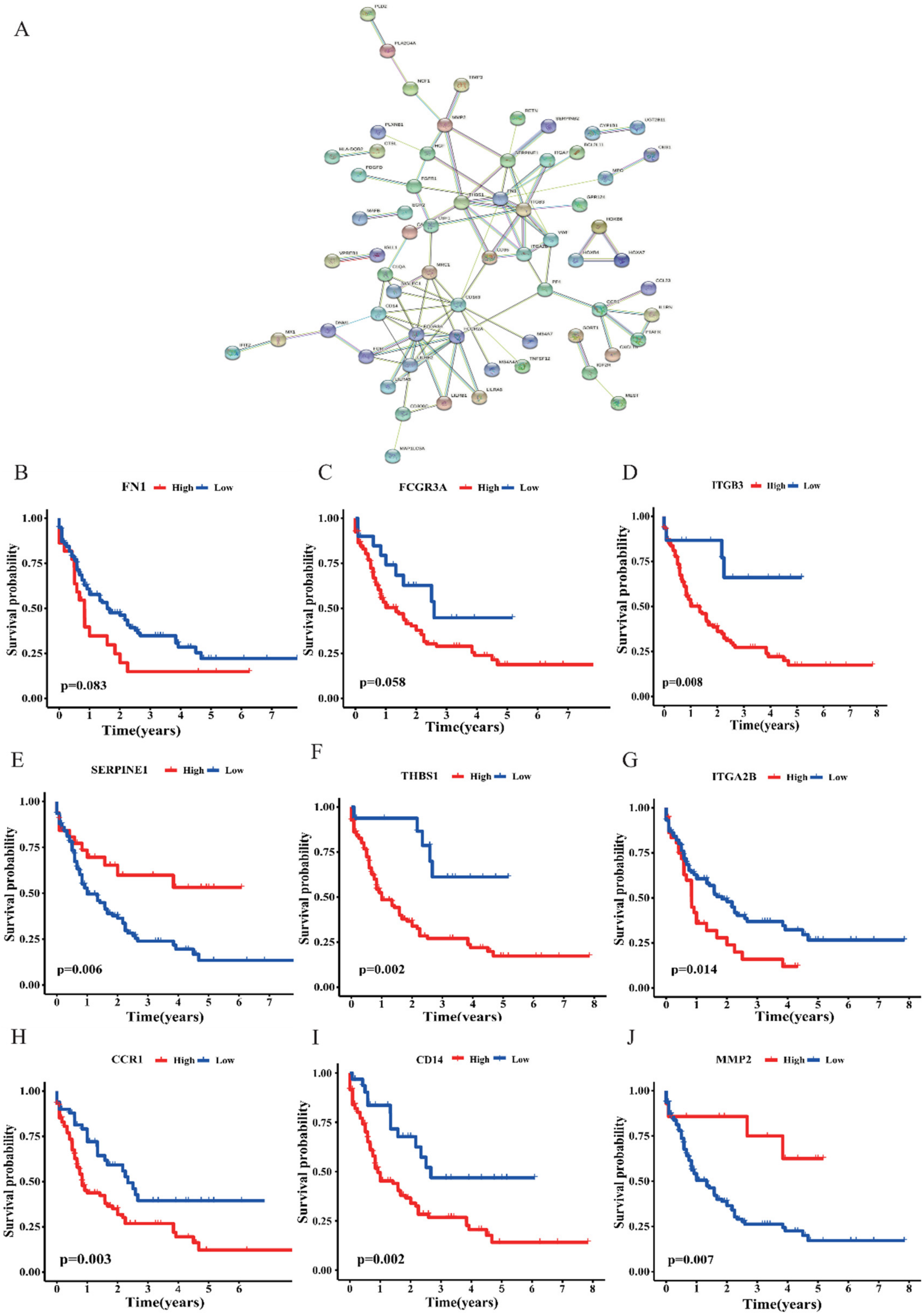

Figure S2. Construction of the protein–protein interaction (PPI) network and prognostic value of the top 10 hub genes. (**A)** PPI network of DEGs between the low- and high-risk score groups. (**B-J**) Survival analysis for AML patients with high or low FN1 (**B**), FCGR3A (**C**), ITGB3 (**D**), SERPINE1 (**E**), THSB1 (**F**), ITGA2B (**G**), CCR1 (**H**), CD14 (**I**), and MMP2 (**J**).

Table S1. The primer sequences of genes.

	Forward Primers	Reverse Primers
UROD	5'- CGACACTTGGAGGTTCAACAATAA-3'	5'- CACCACCCTCAACCATGTATGT-3'
PCTP	5'- CACCACCCTCAACCATGTATGT-3'	5'- CACCACCCTCAACCATGTATGT-3'
PLA2G4A	5'- CACCACCCTCAACCATGTATGT-3'	5'- ATCACTCACCAAGGCCATTATCA-3'
EPHX2	5'- GGATTTCCCGAGAGTTGGTATTC-3'	5'- GCTTGAGAGAGGCCCAGTTTAT-3'
GAPDH	5'-GTCTCCTCTGACTTCAACAGCG-3'	5'-ACCACCCTGTTGCTGTAGCCAA-3'
